# Reduced nicotine dependence following traumatic brain injury in an elderly patient: a case report and literature review

**DOI:** 10.3389/fnhum.2025.1619775

**Published:** 2025-07-17

**Authors:** Min Yuan, Renshi Xu

**Affiliations:** ^1^Department of Neurology, Jiangxi Provincial People's Hospital, The First Affiliated Hospital of Nanchang Medical College, Nanchang, China; ^2^Department of Neurology, Xiangya Hospital, Central South University, Jiangxi Hospital, National Regional Center for Neurological Diseases, Nanchang, China

**Keywords:** traumatic brain injury, nicotine dependence, addiction, elderly, case report

## Abstract

Traumatic brain injury (TBI) can induce a range of neurological and behavioral changes, including potential effects on substance dependence. We present the case of an 87-year-old male with a longstanding history of heavy smoking (~60 pack-years) who demonstrated an abrupt cessation of nicotine craving following a severe TBI involving subdural hemorrhage, contusions, and subarachnoid hemorrhage. Clinical management included supportive therapy for intracranial pressure control and infection management. Nicotine dependence and craving were qualitatively assessed through repeated structured clinical interviews during hospitalization and outpatient follow-up. Remarkably, during the six-month follow-up, the patient remained abstinent without signs of withdrawal or nicotine craving and the use of pharmacological or behavioral interventions. This case highlights a rare but significant phenomenon suggesting that severe brain injury may disrupt mesolimbic dopaminergic circuits, including the ventral tegmental area (VTA) and nucleus accumbens (NAc), central to nicotine-related reward processing. We discuss potential neurobiological mechanisms post-injury, including dopaminergic dysfunction and health behavior adaptation. Further research is needed to elucidate the underlying pathways and clinical implications of TBI-associated changes in addictive behaviors.

## Introduction

Traumatic brain injury (TBI) is defined as an alteration in brain function caused by external force and remains a primary global health concern. According to the World Health Organization, TBI accounts for approximately 600,000–900,000 new cases annually, resulting in significant mortality and disability (Bergquist et al., 2024). Beyond the immediate neurological sequelae, TBI is known to induce behavioral and psychiatric changes, potentially affecting substance use behaviors. Nicotine dependence, driven by complex neurobiological pathways, including the dopaminergic reward system, remains one of the most prevalent forms of addiction worldwide (Kim and Picciotto, 2023). While some studies suggest TBI may exacerbate substance use disorders, others report a reduction in addictive behaviors post-injury, highlighting a complex and poorly understood relationship (Menon et al., 2010; Rawat et al., 2023). In this report, we present a rare case of an elderly patient with a longstanding history of heavy smoking who experienced a notable reduction in nicotine dependence following severe TBI, and we discuss possible underlying mechanisms.

## Case presentation

An 87-year-old man was admitted to the hospital with complaints of headache and vomiting lasting over 8 h following a fall at home. He had a medical history significant for lacunar cerebral infarction for more than 10 years, for which he was receiving clopidogrel for antiplatelet therapy and atorvastatin for lipid regulation. He had no history of diabetes, hypertension, or coronary artery disease. There was no history of previous surgery, trauma, blood transfusion, allergies, or hereditary diseases. However, he had a substantial smoking history, consuming at least 50 cigarettes daily for decades, with minimal alcohol consumption.

At the time of the fall, the patient reported dizziness and loss of balance, landing on the left parietal-occipital region with minor scalp bleeding. He subsequently experienced headaches and two episodes of vomiting but did not lose consciousness or experience seizures. Upon arrival at the hospital, he was conscious. Physical examination revealed scalp bruising and local swelling at the left parietal-occipital region. Pupils were equal and reactive to light (2.5 mm diameter), with free ocular movement, supple neck without stiffness, normal limb motor function, preserved physiological reflexes, and no pathological reflexes.

**Figure 1:**
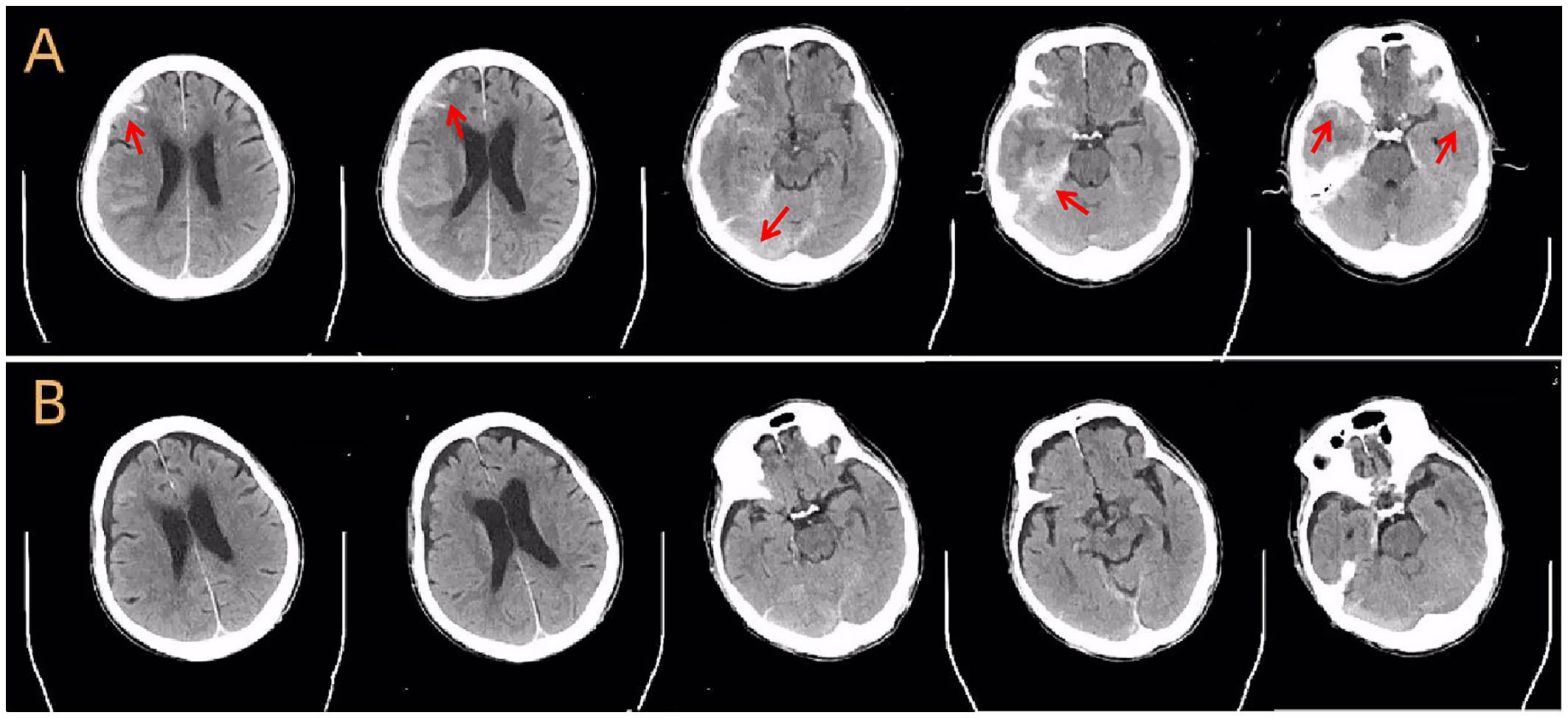
Cranial CT scans before and after treatment. **(A)** Axial non-contrast CT scan at admission shows acute subdural hemorrhage, brain contusion in the frontal and temporal lobes, subarachnoid hemorrhage, and skull fracture (arrows). **(B)** Follow-up CT scan 1 month later shows near-complete resolution of hemorrhage and signs of parenchymal atrophy in the affected regions.

A cranial CT scan demonstrated subdural hemorrhages in the right frontotemporal and left frontotemporal regions, right frontal lobe contusion with hemorrhage, traumatic subarachnoid hemorrhage, fracture of the left parietal bone, and localized scalp soft tissue swelling (Figure 1A).

Further investigations included a pulmonary CT, which revealed bilateral pulmonary infection, a small right pleural effusion, dilated lower lobes of both lungs, emphysema, and thickened pleural adhesions. The electrocardiogram showed arrhythmia with atrial fibrillation. Blood tests indicated leukocytosis (16.61 × 109/L), elevated neutrophil ratio (93.11%), normal hemoglobin (145.4 g/L) and platelet count (150 × 109/L), and mild hypoalbuminemia (albumin 33.7 g/L). Coagulation profiles, liver and kidney function tests, electrolytes, cardiac enzymes, tumor markers, and serologic tests for syphilis, HIV, hepatitis B, and C were negative. Glycated hemoglobin was 6.58%, and random blood glucose was 7.5 mmol/L.

Following admission, the patient was managed with bed rest, debridement, and hemostasis. Based on pulmonary findings, he was administered ceftazidime 2.0 g twice daily for infection control, nimodipine to prevent vasospasm, mannitol 125 mL twice daily for intracranial pressure reduction, and levetiracetam for seizure prophylaxis. Metoprolol sustained-release tablets were prescribed to control ventricular rate. Supportive care included passive limb exercises, lower limb compression therapy for thrombosis prevention, nutritional supplementation with fat emulsions, amino acids, human serum albumin, and fluid management.

During hospitalization, the patient’s headaches gradually improved. Follow-up cranial CT revealed gradual absorption of intracranial hemorrhage, although a small subdural effusion remained (Figure 1B). Management of constipation and gastrointestinal symptoms was accomplished with metoclopramide and passive exercises to promote intestinal peristalsis. Repeat abdominal and pelvic CT scans showed no additional traumatic findings.

After 1 month of intensive treatment, the patient showed significant clinical improvement, resolving headache, nausea, vomiting, cough, and sputum production. Pulmonary infection had resolved on repeat imaging. Functionally, he regained normal mobility.

**Figure 2:**
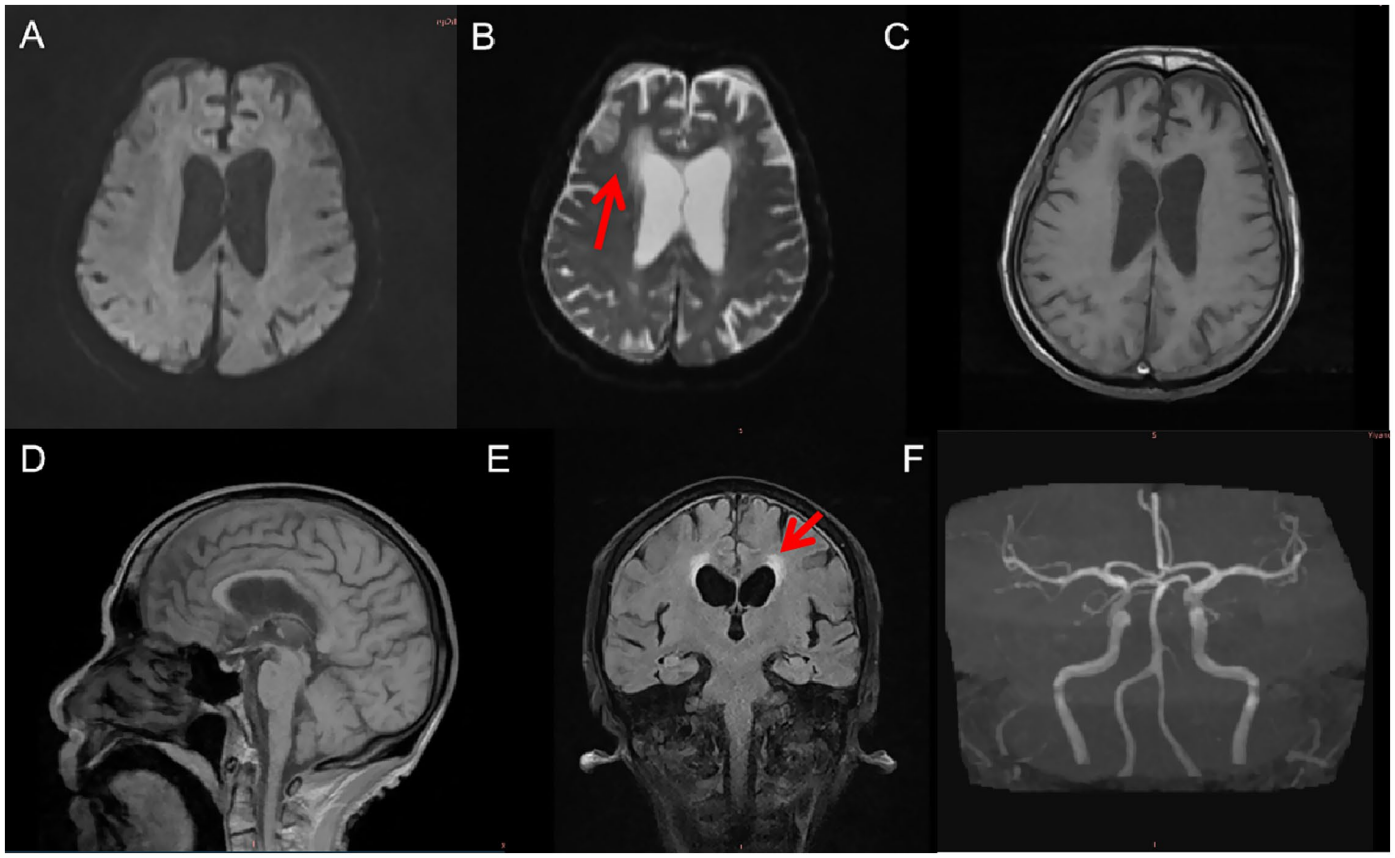
MRI and MRA findings 6 months post-injury. **(A)** Diffusion-weighted imaging (DWI) shows no acute infarction. **(B)** Axial T2-weighted MRI reveals right frontal lobe malacia and mild ventricular enlargement. **(C)** T1-weighted imaging shows cortical atrophy in the right frontal region. **(D)** Sagittal T1-weighted MRI illustrates generalized cerebral atrophy with prominent sulci and ventricles. **(E)** Axial T2-FLAIR imaging demonstrates diffuse leukoaraiosis, especially in the periventricular and deep subcortical white matter. **(F)** MRA reveals multifocal cerebral atherosclerosis.

Remarkably, during the six-month follow-up period, the patient did not report any craving for cigarettes. Cessation occurred without pharmacologic intervention or behavioral support and was not voluntarily initiated by the patient. Throughout hospitalization, no signs of nicotine withdrawal—such as irritability, anxiety, restlessness, or somatic symptoms—were observed by clinical staff or reported by the patient’s family. Daily clinical monitoring and informal interviews with the patient, along with regular discussions with his daughter, consistently revealed an absence of craving or withdrawal symptoms. Although no structured rating scales (e.g., the Fagerström Test for Nicotine Dependence) were administered, the patient’s abstinence was confirmed at 1-, 3-, and 6-month follow-up visits. At each time point, both the patient and his daughter independently reported continued smoking cessation without craving. Cranial MRI revealed right hemispheric malacia, cerebral atrophy, and leukoaraiosis. At the same time, magnetic resonance angiography (MRA) indicated evidence of cerebral atherosclerosis (Figure 2).

The patient is a first-degree relative of the corresponding author. Clinical observations were corroborated by attending physicians and other family members not involved in the study to minimize potential bias.

## Discussion

TBI refers to brain tissue damage caused by external forces, leading to neuronal death or dysfunction. It can result from direct mechanical impact, acceleration-deceleration forces, or intracerebral hemorrhage (Bergquist et al., 2024). The severity of traumatic brain injury can be divided into mild, moderate, and severe levels. Mild brain injury is mainly characterized by transient dizziness or headache; moderate brain injury is characterized by short-term amnesia and disturbance of consciousness; severe brain injury may lead to long-term or permanent neurological dysfunction (Menon et al., 2010). The harm of traumatic brain injury to individuals is multifaceted, including but not limited to the following aspects: (1) impairment of physiological function: Traumatic brain injury will lead to the death and damage of brain cells and then affect the normal function of the brain. This can lead to cognitive impairment, memory loss, poor concentration, and language problems. (2) Emotional and behavioral problems: Traumatic brain injury may trigger emotional and behavioral problems, such as depression, anxiety, irritability, impulsive behavior, etc. These problems can harm an individual’s quality of life and social functioning. (3) Movement disorders: Traumatic brain injury may impair motor function, including muscle weakness, decreased coordination, balance problems, etc. This can lead to problems such as reduced mobility and limb dysfunction (Rawat et al., 2023).

Prior research has reported mixed outcomes regarding substance use following TBI. Some studies suggest increased vulnerability to substance use disorders, particularly in the early post-injury period. For example, Miller et al. (2013) found elevated rates of alcohol and nicotine use within 30 days after mild TBI in active-duty personnel. Ohlmeier et al. (2007) reported higher rates of nicotine and alcohol dependence in individuals with comorbid ADHD following TBI. Conversely, other studies have noted decreased substance use or poorer outcomes associated with pre-injury substance use. Durazzo et al. (2013) observed attenuated neurocognitive recovery in chronic smokers after mild TBI, while Chen et al. (2019) showed that cigarette smoking was associated with worse functional recovery after spontaneous intracerebral hemorrhage. Animal studies, such as that by Verbois et al. (2003), have demonstrated that intermittent nicotine exposure can attenuate cognitive dysfunction after TBI, suggesting complex and bidirectional mechanisms. These heterogeneous findings highlight the influence of lesion characteristics, injury severity, and psychiatric comorbidities in shaping post-TBI substance use trajectories (Table 1).

**Table 1:** Summary of representative studies examining the relationship between traumatic brain injury (TBI) and substance use outcomes.

Study (year)	TBI type	Lesion characteristics	Substance use outcome	Notes
Chen et al. (2019)	Mild TBI	Not specified (military cohort)	↑ Alcohol and nicotine use	Higher substance use within 30 days post-injury in active-duty personnel
Miller et al. (2013)	TBI with ADHD	Not specified	↑ Nicotine and alcohol dependence	Psychiatric comorbidities may increase post-TBI vulnerability
Verbois et al. (2003)	Mild TBI	Not specified	↓ Neurocognitive recovery in smokers	Smoking associated with poorer cognitive outcomes post-TBI
Durazzo et al. (2013)	Spontaneous ICH	Intracerebral hemorrhage	↓ Functional outcomes in smokers	Smoking worsened recovery, relevant to the TBI spectrum
Baumung et al. (2016)	Experimental TBI (rat model)	Cortical/hippocampal damage	↓ Cognitive dysfunction with nicotine	Intermittent nicotine-attenuated TBI-induced deficits
Present Case	Severe TBI	Subdural hemorrhage, contusion, SAH, skull fracture	↓ Nicotine craving post-injury	Complete loss of craving after 50 years of smoking

*TBI, traumatic brain injury; ADHD, attention-deficit/hyperactivity disorder; ICH, intracerebral hemorrhage; ↑ increase; ↓ decrease.

In our case, an elderly patient suffered a severe brain injury involving subdural hemorrhage, contusion, subarachnoid hemorrhage, and skull fracture. Despite a 50-year history of heavy smoking, the patient showed a complete and sustained loss of nicotine craving post-injury, without signs of withdrawal and in the absence of pharmacological or behavioral intervention. This rare clinical observation supports the hypothesis that specific TBI-related lesions may disrupt neural circuits involved in craving and reward regulation. However, the precise neurobiological mechanisms underlying this phenomenon remain to be clarified.

Several hypothetical mechanisms may underlie the reduced nicotine dependence observed in this patient following TBI in elderly patients: (1) Disruption of the brain’s reward system: Brain injuries such as subarachnoid hemorrhage and brain contusion may impair dopaminergic pathways, reducing the reinforcing effects of nicotine and weakening addiction (Baumung et al., 2016). (2) Damage to glutamatergic neurons: TBI may disrupt glutamate transmission, which plays a key role in the excitatory effects of nicotine, thereby reducing craving (Picciotto and Kenny, 2021). (3) Dopaminergic dysfunction: TBI may impair dopamine release, further weakening the reward response to nicotine (Di Chiara and Imperato, 1988). (4) Age-related physical decline: Elderly individuals may already have diminished cardiovascular and respiratory function, reducing their physiological tolerance and psychological dependence on smoking (Wolfe and Gracia, 2023). (5) Increased health awareness post-injury: After experiencing severe brain trauma, elderly patients may become more health-conscious and voluntarily reduce or eliminate smoking behaviors (Li et al., 2021).

Our patient’s spontaneous cessation of smoking following TBI aligns with the hypothesis that disruption of dopaminergic reward pathways—particularly involving the VTA and NAc—may impair the subjective experience of craving. The absence of withdrawal symptoms and lack of external smoking cessation measures further suggest a neurological basis rather than voluntary behavior change. Nonetheless, the complex interplay between injury severity, location, and neurobehavioral outcomes warrants further investigation.

### Limitations

This case report represents a single patient’s experience, limiting the generalizability of the findings. The follow-up duration was relatively short, and we cannot exclude psychosocial factors influencing smoking behavior. Larger prospective studies are needed to substantiate the observed association. While no structured craving or withdrawal scales were employed, the consistency of clinical observations and follow-up interviews supports the likelihood of genuine nicotine abstinence. Nevertheless, the absence of objective behavioral or neuropsychological assessments represents a limitation. Future studies may benefit from incorporating standardized craving and withdrawal instruments to improve reliability.

Additionally, the patient is a first-degree relative of the corresponding author, which may introduce a risk of interpretation bias. Although all clinical data and follow-up observations were verified by medical staff and uninvolved family members, this familial relationship may limit the complete objectivity of the findings. It should be considered when interpreting the results.

## Conclusion

Severe traumatic brain injury may be associated with reduced nicotine dependence, possibly due to disruption of neural reward mechanisms. This case provides valuable clinical insights, but broader studies are essential to clarify the relationship between TBI and addictive behaviors.

## Data Availability

The datasets presented in this article are not readily available because of ethical and privacy restrictions. Requests to access the datasets should be directed to the corresponding author.
